# Cross-cultural analysis of attention disengagement times supports the dissociation of faces and patterns in the infant brain

**DOI:** 10.1038/s41598-019-51034-x

**Published:** 2019-10-08

**Authors:** Juha Pyykkö, Per Ashorn, Ulla Ashorn, Dana J. H. Niehaus, Jukka M. Leppänen

**Affiliations:** 1 Center for Child Health Research, Faculty of Medicine and Health Technology, Tampere University, Tampere, Finland; 2 Department of Paediatrics, Tampere University Hospital, Tampere, Finland; 3 Department of Psychiatry, Faculty of Medicine and Health Sciences, Stellenbosch University, Cape Town, South Africa; 4 Infant Cognition Laboratory, Center for Child Health Research, Faculty of Medicine and Health Technology, Tampere University, Tampere, Finland

**Keywords:** Attention, Saccades, Perception, Pattern vision

## Abstract

Infants are slower to disengage from faces than non-face patterns when distracted by novel competing stimuli. While this perceptual predilection for faces is well documented, its universality and mechanisms in relation to other aspects of attention are poorly understood. We analysed attention disengagement times for faces and non-face patterns in a large sample of 6-to 9-month-old infants (*N* = 637), pooled from eye tracking studies in socioculturally diverse settings (Finland, Malawi, South Africa). Disengagement times were classified into distinct groups of quick and delayed/censored responses by unsupervised clustering. Delayed disengagement was frequent for faces (52.1% of trials), but almost negligible for patterns (3.9% of trials) in all populations. The magnitude of this attentional bias varied by individuals, whereas the impact of situational factors and facial expression was small. Individual variations in disengagement from faces were moderately stable within testing sessions and independent from variations in disengagement times for patterns. These results point to a fundamental dissociation of face and pattern processing in infants and demonstrate that the bias for faces can be robust against distractors and habituation. The results raise the possibility that attention to faces varies as an independent, early-emerging social trait in populations.

## Introduction

Infants from an early age show an attentional bias for faces over patterns or objects^1,2^. Infants are more likely to orient their first eye movements to faces than to other salient objects when seeing a complex scene^3^, dwell longer on faces than non-face objects and patterns^4,5^, and are slower to disengage attention from faces as compared to non-face patterns when “distracted” by a new, competing stimulus in the visual periphery^2^.

Various sources of evidence suggest that infants’ early-emerging attentional bias for faces may reflect a trait that is genetically and neurocognitively distinct from other aspects of attention control in humans^6^. Twin studies show strong and specific genetic variations in different aspects of face processing in children and adults, including the relative weighting of attention to the eye vs. mouth region in toddlers^7,8^, electrocortical responses to facial expressions in adolescents^9^, and recognition of facial identity in adults^10^. Faces and objects elicit differential patterns of electrocortical activation in 6-month-old infants and adults^11,12^, and several studies have documented neurological deficits that affect face processing (e.g., identity recognition) while sparing other visual object recognition capacities^13^. Finally, studies of rare genetic disorders (Williams syndrome) have shown spared face processing in the presence of mild to moderate intellectual disabilities^14–16^.

To examine the dissociation of attentional mechanisms for faces and patterns in infants, we examined covariations in attention disengagement times for faces and non-face patterns in a large sample of infants who were 6 to 9 months old and therefore at the age the bias for faces is clearly evident^5,17,18^. We used unsupervised clustering to identify distinct categories of quick and delayed responses in infant disengagement time data, and subsequently examined the frequencies of these response types for faces and patterns. We also examined whether the bias for faces can be dissociated from other situational variations in infant behaviour (e.g., habituation of disengagement over time, see^19,20^), as well as from more general efficiency and speed of attentional disengagement performance. Contrary to some previous studies^19–21^, the current studies were optimized for studying attention to the faces vs. patterns by including equal presentation probabilities for the two categories, by using patterns that did not have the shape of a face, and by contrasting faces and patterns with maximally salient, lateral “distractors”.

Following Wilmer^22^, we predicted that the independence of the mechanisms mediating attention to faces and patterns results in relatively strong covariation of disengagement times for distinct exemplars of faces *and* relatively lower correlation as well as distinct distribution of disengagement times for faces and patterns. This hypothesis was contrasted with the alternative model predicting overlap in the development of attention disengagement mechanisms for faces and other stimuli^23^ as well as significant covariance in disengagement times for faces and patterns (cf.^24^).

The data for the current analysis were pooled from separate eye tracking studies conducted in Finland, Malawi, and South Africa. The data were collected by using the same methods, but the age and the living environments of the participants (e.g., level of urbanization) varied across sites. While these differences precluded us from performing direct comparisons of populations, it provided a unique opportunity to examine the generalizability of the dissociation of face and pattern processing across heterogeneous samples of infants and while including populations that have traditionally been underrepresented in developmental sciences^25^.

## Results

### Datasets and the distribution of disengagement times

**Figure 1:**
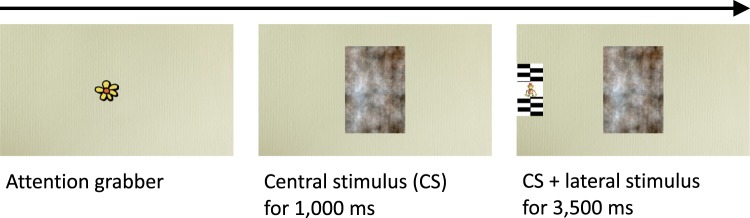
Attention disengagement paradigm. A face or a non-face pattern was presented in the centre of the screen. A lateral stimulus (distractor) was added to the right or to the left of the central stimulus after 1,000 ms. The central stimulus was presented until the end of each trial, thus, overlapping in time with the lateral stimulus.

Infants (*N* = 637) were tested with a paradigm (Fig. 1) that assesses attentional disengagement time (DT) from a centrally presented stimulus (face or pattern) toward the location of a new stimulus in the visual periphery (salient pattern and animation). The data comprised a total of 12,035 valid trials (Table 1). A slightly higher percentage of the valid data were face trials (55.6%) than non-face trials.

**Table 1:** Trials and disengagement times (DTs) for the datasets.

	Finland		Malawi 1		Malawi 2		South Africa	
	Non-faces	Faces	Non-faces	Faces	Non-faces	Faces	Non-faces	Faces
*N* of trials	624	624	576	577	6,064	6,068	2,675	2,677
*N* (%) of valid trials	381 (61.1)	450 (72.1)	333 (57.8)	395 (68.5)	3,478 (57.4)	4,167 (68.7)	1,158 (43.3)	1,673 (62.5)
Proportion of quick DTs, %	97.4	72.2	96.1	43.8	96.9	38.7	93.4	65.4
Proportion of delayed DTs, %	2.1	14.2	3.6	17.5	2.8	30.5	4.7	13.3
Proportion of censored DTs (≥3500 ms), %	0.5	13.6	0.3	38.7	0.3	30.8	2.0	21.3
Mean (*SD*) of quick DTs, ms	348 (141)	426 (177)	340 (134)	438 (157)	353 (147)	475 (189)	373 (161)	435 (160)

**Figure 2:**
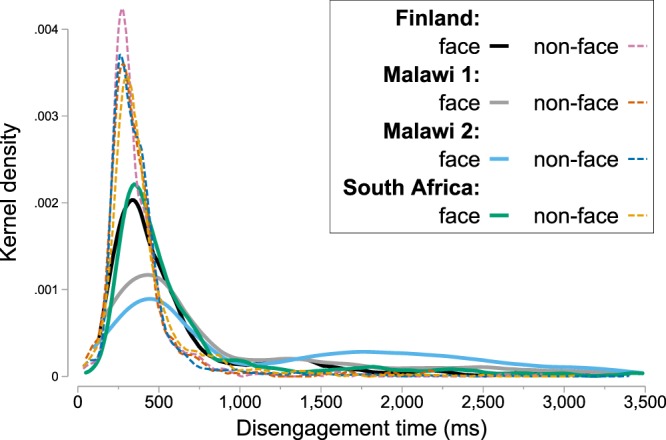
Distributions of disengagement times for faces and non-face patterns as kernel densities for disengagement times below 3,500 ms. Censored disengagement times (i.e., disengagement was not observed by 3,500 ms) are not shown.

The DTs on the valid trials compromised a heterogeneous distribution of quick, delayed, and censored values (Fig. 2). To summarize this distribution for individual infants, we used a data-driven clustering method to recode the DT data into a binary variable that indicated whether a quick disengagement from the central stimulus occurred or not on a given trial (Supplementary Methods). Based on this analysis, 30.6% of the valid trials were trials on which the DT from the central to the lateral stimulus was *delayed* (i.e., the latency of the disengagement was outside the range of typical disengagement latencies or disengagement was not observed by the end of the trial period).

### Factors underlying delayed disengagement

Our first analyses used a conditional fixed-effects logistic regression model and the data from all valid trials to examine how infants’ attention disengagement from the central to the lateral stimulus was affected by the content of the central stimulus (face vs. non-face), the previous trial, the lateral stimulus, and the trial sequence. This analysis showed that the probability of delayed disengagement was most strongly affected by the category of the central stimulus, as odds ratios varied between 10.49 and 86.65 for delayed disengagement when a face was presented compared to a non-face pattern (Table 2). Other covariates’ odds ratios for delayed disengagement varied between 0.57 and 1.66 across datasets.

**Table 2:** Conditional fixed-effects logistic regression model for delayed disengagement with both face and non-face trials included for each dataset.

Delayed disengagement	Finland, *N* = 681 DTs, 30 participants, Pseudo *R*^2^ = 0.25		Malawi 1, *N* = 715 DTs, 35 participants, Pseudo *R*^2^ = 0.37		Malawi 2, *N* = 7,442 DTs, 358 participants, Pseudo *R*^2^ = 0.46		South Africa, *N* = 2,600 DTs, 147 participants, Pseudo *R*^2^ = 0.18	
	Odds ratio (95% CI)	*P*	Odds ratio (95% CI)	*P*	Odds ratio (95% CI)	*P*	Odds ratio (95% CI)	*P*
Trial was face instead of non-face pattern	18.60 (9.12, 37.93)	<0.001	39.45 (21.02, 74.04)	<0.001	86.65 (68.49, 109.63)	<0.001	10.49 (7.93, 13.88)	<0.001
Previous trial was invalid	1.15 (0.64, 2.09)	0.64	1.14 (0.69, 1.88)	0.60	1.15 (0.97, 1.37)	0.12	0.94 (0.74, 1.20)	0.63
Lateral stimulus was on the left	0.86 (0.55, 1.34)	0.51	0.91 (0.61, 1.36)	0.66	0.76 (0.67, 0.87)	0.003	1.12 (0.91, 1.37)	0.29
First face of a block	0.57 (0.14, 2.22)	0.41	0.77 (0.29, 2.03)	0.60	1.61 (1.18, 2.21)	<0.001	0.73 (0.45, 1.21)	0.23
Trial number in a block	0.97 (0.87, 1.07)	0.56	0.90 (0.81, 0.99)	0.03	0.94 (0.91, 0.97)	<0.001	0.99 (0.94, 1.04)	0.64
Block:								
1	1.00	N/A	1.00	N/A	1.00	N/A	1.00	N/A
2	1.36 (0.76, 2.44)	0.30	0.94 (0.54, 1.62)	0.54	1.10 (0.92, 1.33)	0.31	0.73 (0.55, 0.97)	0.03
3	0.72 (0.38, 1.36)	0.31	0.88 (0.51, 1.54)	0.51	1.10 (0.91, 1.32)	0.32	0.84 (0.63, 1.12)	0.23
4	0.86 (0.45, 1.63)	0.64	0.95 (0.53, 1.71)	0.53	1.66 (1.36, 2.02)	<0.001	0.79 (0.58, 1.08)	0.14

**Figure 3:**
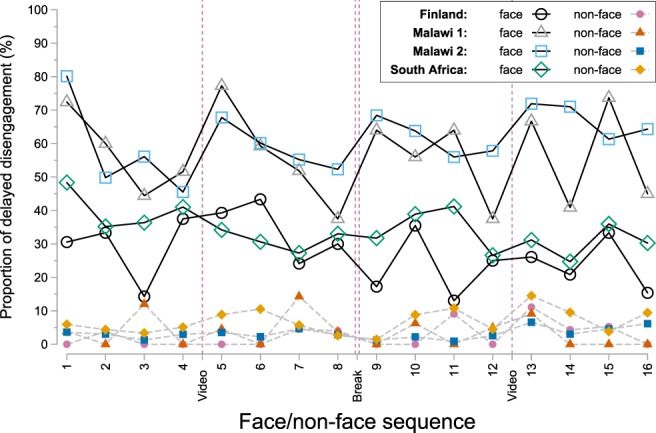
Proportion of delayed disengagement from faces and non-face patterns as a function of block and trial. Vertical dashed lines indicate breaks between blocks.

Delayed disengagement was frequent for faces (52.1% of all trials, 27.8–61.3% across datasets) throughout the testing session, but very rare for non-face patterns (3.9% of all trials, 2.6–6.6% across datasets) (Fig. 3). Illustrations of the differences in infants’ disengagement from faces and patterns are provided in a Supplementary Video.

Our second logistic regression analysis focused on trials with a face stimulus to examine whether delayed disengagement from faces was affected by the facial expression (happy vs. fearful), the identity (face model A vs. B), the disengagement response on the previous trial (quick vs. delayed disengagement), the trial being the first face of the block, the face sequence in the block (1–4), and the trial block (1–4). This analysis showed a consistent, although relatively small effect of the preceding stimulus, the facial expression, and the facial identity (Table 3). The effect of the previous trial was explained by an increase in delayed disengagement from faces if the stimulus in the preceding trial was a non-face pattern (i.e., if the previous trial was a face, odds ratios for delayed disengagement varied between 0.36–0.85 depending on the previous response type). The likelihood of delayed disengagement was also higher for fearful as compared to happy faces (odds ratios 1.35–2.13 across datasets). Other covariates’ odds ratios varied between 0.52 and 1.66 across datasets.

**Table 3:** Conditional fixed-effects logistic regression model for delayed disengagement with only face trials included for each dataset.

Delayed disengagement	Finland, *N* = 367 DTs, 30 participants, Pseudo *R*^2^ = 0.05		Malawi 1, *N* = 373 DTs, 33 participants, Pseudo *R*^2^ = 0.11		Malawi 2, *N* = 3,850 DTs, 330 participants, Pseudo *R*^2^ = 0.06		South Africa, *N* = 1,483 DTs, 140 participants, Pseudo *R*^2^ = 0.03	
	Odds ratio (95% CI)	*P*	Odds ratio (95% CI)	*P*	Odds ratio (95% CI)	*P*	Odds ratio (95% CI)	*P*
Face was fearful instead of happy	1.61 (1.00, 2.60)	0.05	2.13 (1.33, 3.41)	0.002	1.35 (1.17, 1.56)	<0.001	1.51 (1.19, 1.92)	0.001
Face identity was A instead of B	0.58 (0.34, 0.99)	0.05	0.52 (0.32, 0.84)	0.007	0.64 (0.55, 0.74)	<0.001	N/A	N/A
Previous trial was:								
Non-face pattern	1.00	N/A	1.00	N/A	1.00	N/A	1.00	N/A
Face and quick disengagement	0.74 (0.41, 1.36)	0.33	0.85 (0.46, 1.58)	0.61	0.75 (0.61, 0.92)	0.006	0.74 (0.55, 0.99)	0.05
Face and delayed disengagement	0.80 (0.38, 1.70)	0.57	0.36 (0.19, 0.69)	0.002	0.54 (0.44, 0.65)	<0.001	0.68 (0.47, 0.98)	0.04
First face of a block	0.67 (0.27, 1.66)	0.39	0.99 (0.41, 2.37)	0.98	1.45 (1.11, 1.91)	0.007	1.19 (0.77, 1.85)	0.44
Face sequence in a block	0.81 (0.57, 1.13)	0.22	0.69 (0.50, 0.95)	0.02	0.87 (0.78, 0.96)	0.006	1.01 (0.85, 1.20)	0.92
Block:								
1	1.00	N/A	1.00	N/A	1.00	N/A	1.00	N/A
2	1.09 (0.56, 2.13)	0.80	0.74 (0.39, 1.39)	0.35	1.09 (0.89, 1.33)	0.41	0.65 (0.47, 0.89)	0.008
3	0.61 (0.31, 1.21)	0.16	0.79 (0.42, 1.47)	0.46	1.22 (1.00, 1.49)	0.05	0.77 (0.56, 1.07)	0.12
4	0.54 (0.26, 1.13)	0.10	0.86 (0.43, 1.70)	0.66	1.66 (1.34, 2.06)	<0.001	0.63 (0.45, 0.90)	0.01

### Delayed disengagement and general oculomotor speed

**Figure 4:**
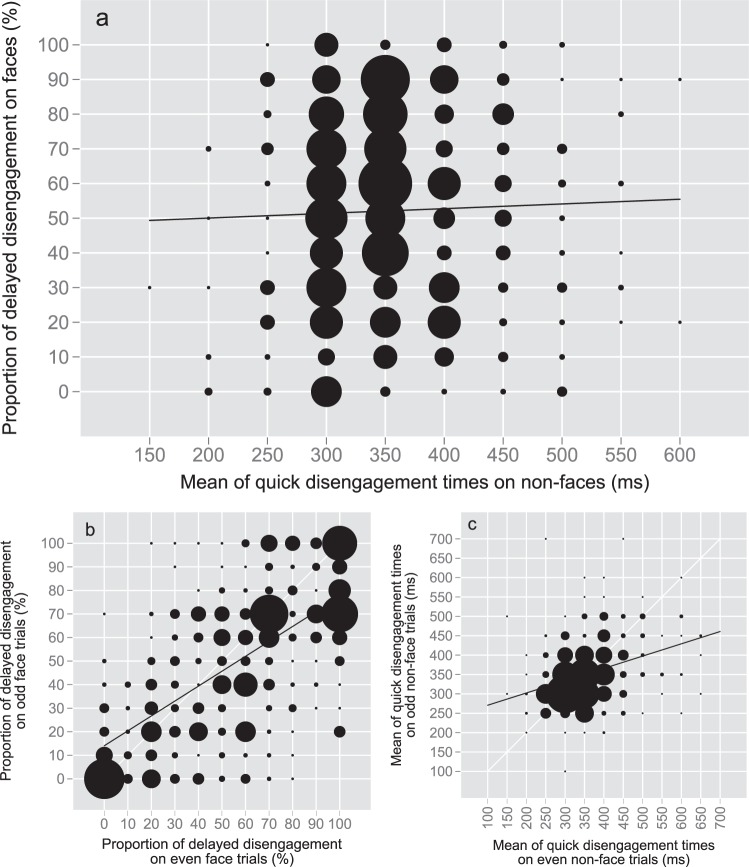
Bubble plots on disengagement data for all studies pooled including participants with ≥3 observations for each variable. Circle size is the squared count of participants for each interval. Black line indicates linear fit. (**a**) Covariation of delayed disengagement from faces and oculomotor speed for non-face patterns, *N* = 550, *r*_*s*_ = 0.04, *BF*_10_ = 0.09. (**b**) Odd-even split-half reliabilities of delayed disengagement from faces, *N* = 508, *r*_*s*_ = 0.64, *BF*_10_ > 100. (**c**) Odd-even split-half reliabilities of oculomotor speed for non-face patterns, *N* = 408, *r*_*s*_ = 0.34, *BF*_10_ > 100.

We next examined whether variations in delayed disengagement from faces were associated with more general variations in attentional disengagement or oculomotor speed, as assessed by the mean of DTs for non-faces (Fig. 4). In an analysis pooling data from the four datasets, this correlation was 0.04 (*BF*_10_ = 0.09), indicating that 0.2% of the variance in delayed disengagement was explained by more general variations in oculomotor speed. In comparison, the correlation in delayed disengagement between fearful and happy faces was 0.66 (*BF*_10_ > 100).

To assess the impact of measurement noise on the correlation, Spearman-Brown corrected correlation between the probability of delayed disengagement and oculomotor speed was 0.08. Reliability indices for the probability of delayed disengagement and oculomotor speed as Spearman-Brown corrected odd-even split-half correlations were 0.78 and 0.50, respectively.

**Figure 5:**
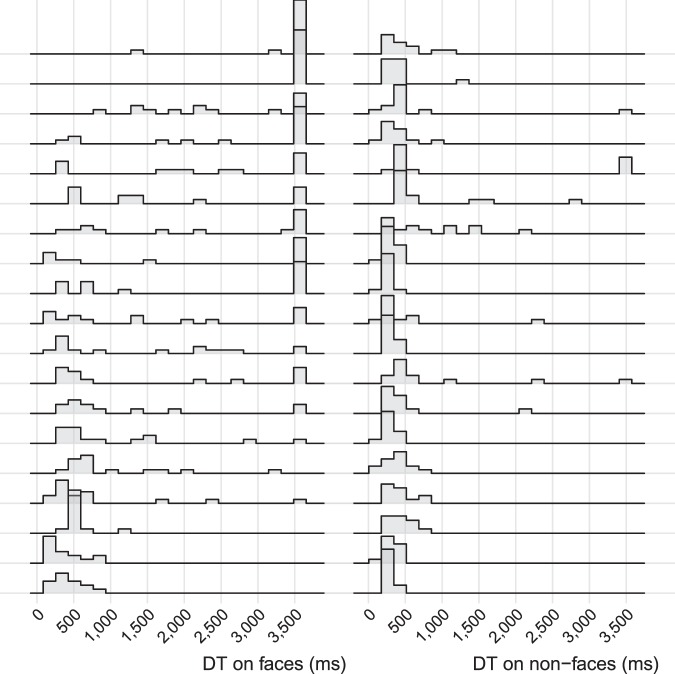
Illustration of disengagement times (DTs) for selected 19 participants with a varying range of delayed disengagement from faces. Each line represents one participant’s histogram distribution of DTs on faces and non-face patterns (bandwidth = 170 ms).

The low correlation and dissociation of the DTs for faces and non-face patterns in individual infants is further illustrated in Fig. 5. As shown by this figure, the relatively drastic individual variations in disengagement from faces were not accompanied by similar variations in disengagement from patterns. In other words, individual infants with high probability of delayed disengagement from faces exhibited typical DTs for patterns.

Given that the pooled analysis of correlations is sensitive to population-level variations (e.g., populations with high probability of delayed disengagement may have slower oculomotor speed), and the possibility that population-level variations mask within-population associations, we replicated the correlation analyses for each dataset separately (Supplementary Figs S1–S4). Correlations between the probability of delayed disengagement and oculomotor speed were 0.37 (*BF*_10_ = 2.30) for Finland, −0.02 (*BF*_10_ = 0.21) for Malawi 1, 0.08 (*BF*_10_ = 0.18) for Malawi 2, and 0.08 (*BF*_10_ = 0.16) for South Africa.

## Discussion

The present analysis of data from a pooled and diverse sample of 637 infants showed two main results that extend our understanding of infants’ attentional bias for faces. First, the result showed unequivocally that delayed disengagement of attention (i.e., gaze shift from an old to a new stimulus, defined here by using a data-driven clustering) is relatively frequent for faces but nearly absent for non-face patterns. This fundamental difference in attention to faces and patterns was clearly evident in all populations, although there were differences in magnitude of the difference between the study sites. Second, our analyses showed that individual variations in the probability of delayed disengagement from faces were not associated with variations in a closely similar measure reflecting the speed of attention disengagement from a non-face pattern, pointing to a clear dissociation of face and pattern processing in infants.

The current analysis provides a comprehensive modelling of the trial-by-trial variability in infants’ attention to faces and non-faces in a design that was optimized for examining this distinction without confounding factors (i.e., faces and patterns were presented with an equal probability, differed in shape, and attention to faces/patterns was contrasted with salient “distractor” stimuli). In addition to showing that infants prioritize attention to faces over patterns, our data showed that disengagement times are also subject to small, but systematic trial-by-trial and stimulus-related variations. The probability of a delayed response was higher when a non-face trial preceded a face trial. There was also evidence for a discontinuity of delayed response over successive trials (i.e., a delayed response decreasing the probability of a delayed response in the next trial). Finally, the likelihood of a delayed response was higher for fearful as compared to happy faces. These variations are likely to be attributable to infants’ well-established tendency to dishabituate to stimulus changes^26^ as well as infants’ apparently universal attentional bias for fearful faces as a physically distinct, novel, and potentially affectively significant social signal^27–33^.

Critically, the probability of delayed disengagement was most strongly predicted by the stimulus category (i.e., higher for faces vs. non-faces), and compared to this effect, the trial-by-trial variations in the likelihood of this response, as well as the difference for faces displaying fearful vs. happy expressions had little explanatory power. It is, therefore, possible that the delayed disengagement from faces is primarily explained by a broadly tuned and potentially universal bias for faces in infants^1^, that is relatively robust against situational variations in infant attention (e.g., habituation) and variations in facial expression. The differences in the point estimates of disengagement probability between the study sites may reflect true population differences in the magnitude of the attentional bias for faces, but could not be meaningfully analysed in the present data given uncontrolled heterogeneity in the samples (e.g., age, urbanization).

One limitation of the current analysis is that only one class of non-face objects was used, and while the faces and patterns were matched in terms of low-level physical properties, the patterns were not recognizable as meaningful objects^21^. It is therefore not known, whether the negligible proportion of delayed disengagement generalizes to other non-face object categories, and in particular, identifiable patterns. The plausibility of this prediction is suggested by findings showing that there is a large difference between attentional dwell times for faces and objects, whereas the differences in infants’ dwell times for different categories of non-face patterns, such as scrambled faces and non-face objects (e.g., picture of animals, body parts, etc.), appear relatively small^5,34^. This suggests that the type of the non-face object may not be a critical factor.

As a second main finding, the current analyses showed that the probability of delayed disengagement from faces varied noticeably between individuals, and that these variations were independent from other similar measures of attention. Previous analyses of data from one of the present datasets as well as other datasets have shown that variations in disengagement times have moderate to high within-session as well as test-retest stability^35–38^, suggesting that they may reflect relatively stable traits in infant behaviour. Importantly, our current results further showed that these variations were only weakly associated with differences in oculomotor speed, as measured by the mean latency of gaze disengagement from non-face patterns. While this result does not prove the specificity of the observed variations for faces, it is consistent with this possibility, and shows a dissociation of two proximally related behaviours (i.e., lack of correlation between two similar measures). Such dissociation is a hallmark of neurocognitively dissociable traits^22^.

The correlation between delayed disengagement from faces and oculomotor speed remains low when this correlation is corrected for the unreliability of its constituent variable (i.e., low reliability estimates), suggesting that variability in delayed disengagement is not associated with individual variations in oculomotor speed. Together, our results are therefore consistent with the possibility that attention to faces is a neurocognitively dissociable trait that varies in strength within populations. The origins (e.g.^7^) of this variability may provide important insights into early social development in infants, given preliminary evidence linking this bias with early social development^21,39^.

## Methods

### Participants

The data for this article include a total of 637 participants, pooled from a study comparing Finnish and Malawian infants^40^, a prospective longitudinal study in Malawi^37^, as well as an unpublished study conducted in South Africa. Infants were assessed at the age of 9 months in Finland (*N* = 39, mean (*SD*) age 274 (4) days, 51% females, all Caucasian), Malawi 1 (*N* = 37, mean (*SD*) age 274 (6) days, 48% females, all Black) and Malawi 2 (*N* = 389, mean (*SD*) age 275 (4) days, 50% females, all Black) studies, and at 6 months in South Africa (*N* = 172, mean (*SD*) age 190 (17) days, 38% females, 22% Black, 26% Coloured, 52% Caucasian). Participants were recruited through a population information system from families residing in urban settings in Finland, from rural villages in Malawi, or from a private well-baby clinic and a Maternal Mental Health Outpatient Clinic in the Cape Town metropolitan area, South Africa.

The studies were conducted in accordance with the ethical standards of the Helsinki declaration. The studies were approved by the institutional review boards of Tampere University; College of Medicine, University of Malawi; and Stellenbosch University. A written informed consent was obtained from a parent or legal guardian on behalf of the participants.

### Eye tracking assessment

The studies used the same eye tracking paradigms to assess attention disengagement, visual search, and sequence learning abilities (as described in^37,40^). The eye tracking data for this article come from the attention disengagement or “overlap” task used to assess infants’ attention to a central stimulus (face or pattern) when a lateral distractor stimulus was presented to compete with the infant’s interest^41–43^. The data from the other tests were not used as they did not assess attention disengagement, or did not include comparable data from face and non-face conditions.

#### Setting and equipment

Infants were seated on their mother’s lap in a baby carrier so that the infant’s eyes were at an approximately 60 cm viewing distance from a 22-inch widescreen monitor with a Tobii X2-60 or T60 eye tracker (Tobii Technology, Stockholm, Sweden). After being positioned in front of the eye tracker, infants watched a sequence of visual stimuli on the screen, presented by using a custom-written MATLAB^44^ script and Psychtoolbox^45^. The stimulus presentation computer communicated with the eye tracking hardware via a Tobii SDK plug-in.

#### Calibration and tests

The assessment started with a 5-point calibration. During the calibration, a cartoon figure (4°) with accompanying sounds was presented consecutively in each corner and in the centre of the screen. Based on the experimenter’s comparison of the calibration outcome with a predefined standard for an acceptable calibration, the calibration was accepted after the first round of calibration or repeated up to two times for each participant.

The calibration was followed by three different eye tracking tests (attention disengagement, visual search, and sequence learning). The three tests were performed twice during the visit by each infant in two separate sessions, with a break in between the sessions (for further details, see^37,40^).

#### Attention disengagement paradigm

Each trial in the test assessing disengagement times for faces and non-faces started with a dynamic attention-grabbing stimulus presented on the centre of the screen (Fig. 1). After the infant fixated on the stimulus (i.e., infant’s point of gaze entered a predefined area of interest in the centre of the screen), a face/pattern and a lateral “distractor” were presented with a 1,000 ms onset asynchrony. The face/pattern was presented on the centre of the screen. The lateral stimulus was presented to the left or right side of the screen (i.e., an overlap) so that the furthest edge of lateral stimulus bordered the edge of the screen (22° away from the centre).

The face stimulus was a picture of a face displaying a happy or fearful expression (the skin colour/ethnicity of the face was matched with the skin colour/ethnicity of the child). Happy and fearful expressions were chosen to assess the impact of facial expression and based on previous results showing that iinfants at this age exhibit differences in disengagement from happy and fearful expressions^31^. In Finland and Malawi, infants saw happy and fearful faces of two matched female models. In South Africa, infants saw happy and fearful faces of one matched female model. The non-face patterns were created by randomizing the phase-spectrum of the face images so that the pattern retains the colour and amplitude spectrum of the original images, but not a shape of the face or any recognizable features of a face. The lateral stimulus was a geometric shape (black and white circles or a checkerboard pattern), which was superimposed by a still picture showing the first frame of a child-friendly cartoon animation.

When the infant shifted gaze to the lateral image, the still picture turned into a dynamic cartoon animation that played for up to 4,000 ms. This combination of a salient low-level stimulus (checkerboard pattern) and a dynamic cartoon was chosen to maximize the probability of attention disengagement from the central to the lateral stimulus, and on the basis of previous data showing that the typical habituation of disengagement over the course of the experiment^19,20^ can be avoided by using dynamic lateral stimuli^36^.

Infants saw a total of 16 face (eight happy and eight fearful) and 16 non-face trials, divided into four blocks of eight trials (i.e., two happy faces, two fearful faces, and four non-faces from one female). The stimuli were presented in a random order within each block. Stimulus blocks 1 and 2 were separated by a short video, blocks 2 and 3 by other eye tracking tests as well as a structured observation (resulting in a longer break), and blocks 3 and 4 again with a short video.

### Data reduction and analysis

Eye tracking time series (*xy*-coordinates of eye positions, 60 Hz) were pre-processed and analysed by using a library of MATLAB functions^36^. The *xy*-coordinates corresponding to the two eyes were merged by taking a mean of the coordinates (or by using the eye with valid *xy*-coordinates if the other eye’s coordinates were invalid), extrapolated to fill missing data points (maximum of 200 ms), and median filtered with a nine-sample (150 ms) moving window to remove abrupt technical spike artefacts from the data. Trials that violated the upper limit of extrapolation (200 ms), had less than 70% fixation on the central stimulus prior to attention shift, and trials on which the shift occurred during a period of extrapolated data were excluded.

The disengagement time (DT) was defined as the time interval starting at the onset of the lateral stimulus and extending until the point of gaze shifted from the central to the lateral stimulus or a time-out period of 3,500 ms was reached (i.e., censored DTs). Given the fact that infants’ DTs are characterized by a heterogeneous distribution of quick, delayed and censored values^37,46^ (Fig. 2), we used a data-driven clustering method to recode DT data into a binary variable that indicated whether a quick disengagement from the central stimulus occurred or not on a given trial (see Supplementary Methods for a detailed description).

In the first set of statistical analysis, we used the conditional fixed-effects logistic regression model in Stata 15.1^47^ to estimate the effects of stimulus- and situational variables on attention disengagement in infants. Accordingly, the first model included the stimulus (face vs. non-face), the validity on the previous trial (valid vs. invalid), the lateral stimulus side (left vs. right), the first face of the block, the trial number in the block (1–8), and the trial block (1–4) as predictors, and the binary classification of attention disengagement (i.e., quick vs. delayed disengagement) as a dependent variable. The second model focused on trials with a face stimulus and examined the effect of the facial expression (happy vs. fearful), the identity (face model A vs. B), the disengagement response on the previous trial (quick vs. delayed disengagement), the first face of the block, the face sequence in the block (1–4), and the trial block (1–4). Based on the method’s requirements, all participants with variability in the dependent variable were included in models as panel variables (i.e., measuring a within-participant variation as participants with all quick or all delayed responses were not included). The models were done for each dataset separately. The method does not estimate a constant term for models.

In the second set of analyses, we tested the hypothesis that the probability of attention disengagement from faces is associated with more general variations in attention disengagement or oculomotor speed (i.e., the disengagement probability is higher for individuals with relatively faster DTs for non-face patterns). For this analysis, we calculated Spearman correlations coefficients (*r*_*s*_) and Bayes factors (*BF*_10_) between the average probability of delayed disengagements for faces and mean of quick DTs for non-face patterns. Bayes factors were calculated with JASP 0.10.2.0^48^ using the default null hypothesis with a uniform prior distribution for *r*_*s*_^49^. Also, we calculated odd-even split-half Spearman correlation coefficients for probabilities of delayed disengagement from faces and for quick DTs for non-face patterns, respectively. To estimate the impact of measurement noise on these correlations, we calculated reliability estimates for the variables as Spearman-Brown corrected correlations. Based on previous studies (e.g.^21^), three or more observations were required for each variable from a participant to be included in a respective correlation coefficient analysis.

## Supplementary information

Supplementary Information

Supplementary Video

## Data Availability

The datasets are be available from the corresponding author on reasonable request.

## References

[1] Johnson MH (2005). Subcortical face processing. Nat. Rev. Neurosci..

[2] Leppänen JM (2016). Using eye tracking to understand infants’ attentional bias for faces. Child Dev. Perspectives.

[3] Kelly, D. J., Duarte, S., Meary, D., Bindemann, M. & Pascalis, O. Infants rapidly detect human faces in complex naturalistic visual scenes. *Dev. Sci*. e12829, 10.1111/desc.12829 (2019). 10.1111/desc.12829 30896078

[4] Amso D, Haas S, Markant J (2014). An eye tracking investigation of developmental change in bottom-up attention orienting to faces in cluttered natural scenes. PLoS One.

[5] Gluckman M, Johnson SP (2013). Attentional capture by social stimuli in young infants. Front. Psychol..

[6] Wilmer, J. B., Germine, L. T. & Nakayama, K. Face recognition: a model specific ability. *Front. Hum. Neurosci*. **8**, 10.3389/fnhum.2014.00769 (2014). 10.3389/fnhum.2014.00769 PMC4193262 25346673

[7] Constantino JN (2017). Infant viewing of social scenes is under genetic control and is atypical in autism. Nat..

[8] Kennedy DP (2017). Genetic influence on eye movements to complex scenes at short timescales. Curr. Biol..

[9] Anokhin A, Golosheykin S, Heath A (2010). Heritability of individual differences in cortical processing of facial affect. Behav. Genet..

[10] Wilmer JB (2010). Human face recognition ability is specific and highly heritable. Proc. Natl. Acad. Sci. United States Am..

[11] de Haan M, Nelson CA (1999). Brain activity differentiates face and object processing in 6-month-old infants. Dev. Psychol..

[12] Halit H, Csibra G, Volein Á, Johnson M. H. (2004). Face-sensitive cortical processing in early infancy. J. Child Psychol. Psychiatry.

[13] Duchaine BC, Nakayama K (2006). Developmental prosopagnosia: a window to content-specific face processing. Curr. Opin. Neurobiol..

[14] Asada K, Itakura S (2012). Social phenotypes of autism spectrum disorders and Williams syndrome: similarities and differences. Front. Psychol..

[15] Ibernon, L., Touchet, C. & Pochon, R. Emotion recognition as a real strength in Williams syndrome: evidence from a dynamic non-verbal task. *Front. Psychol*. **9**, 10.3389/fpsyg.2018.00463 (2018). 10.3389/fpsyg.2018.00463 PMC5895718 29674990

[16] Riby DM, Hancock PJB (2008). Viewing it differently: social scene perception in Williams syndrome and autism. Neuropsychol..

[17] Matsuzawa M, Shimojo S (1997). Infants’ fast saccades in the gap paradigm and development of visual attention. Infant Behav. Dev..

[18] Hunnius S, Geuze RH, van Geert P (2006). Associations between the developmental trajectories of visual scanning and disengagement of attention in infants. Infant Behav. Dev..

[19] Kataja, E.-L. *et al*. Maternal depressive symptoms during the pre- and postnatal periods and infant attention to emotional faces. *Child Dev*., 10.1111/cdev.13152 (2018). 10.1111/cdev.13152 30295323

[20] Leppänen JM (2011). Serotonin and early cognitive development: variation in the tryptophan hydroxylase 2 gene is associated with visual attention in 7-month-old infants. J. Child Psychol. Psychiatry.

[21] Peltola Mikko J., Yrttiaho Santeri, Leppänen Jukka M. (2018). Infants' attention bias to faces as an early marker of social development. Developmental Science.

[22] Wilmer J (2008). How to use individual differences to isolate functional organization, biology, and utility of visual functions; with illustrative proposals for stereopsis. Spatial Vis..

[23] Hood BM, Willen JD, Driver J (1998). Adult’s eyes trigger shifts of visual attention in human infants. Psychol. Sci..

[24] Frank MC, Amso D, Johnson SP (2014). Visual search and attention to faces during early infancy. J. Exp. Child Psychol..

[25] Nielsen M, Haun D, Kärtner J, Legare CH (2017). The persistent sampling bias in developmental psychology: a call to action. J. Exp. Child Psychol..

[26] Colombo J, Mitchell DW (2009). Infant visual habituation. Neurobiol. Learn. Mem..

[27] Conejero Á, Rueda MR (2018). Infant temperament and family socio-economic status in relation to the emergence of attention regulation. Sci. Rep..

[28] Geangu E (2016). Culture shapes 7-month-olds’ perceptual strategies in discriminating facial expressions of emotion. Curr. Biol..

[29] Leppänen JM, Nelson CA (2012). Early development of fear processing. Curr. Dir. Psychol. Sci..

[30] Nakagawa A, Sukigara M (2019). Attentional bias assessed by a facial expression cuing paradigm in infants. Sci. Rep..

[31] Peltola MJ, Leppänen JM, Palokangas T, Hietanen JK (2008). Fearful faces modulate looking duration and attention disengagement in 7-month-old infants. Dev. Sci..

[32] Peltola MJ, Leppänen JM, Vogel-Farley VK, Hietanen JK, Nelson CA (2009). Fearful faces but not fearful eyes alone delay attention disengagement in 7-month-old infants. Emot..

[33] Somerville LH, Whalen PJ (2006). Prior experience as a stimulus category confound: an example using facial expressions of emotion. Soc. Cogn. Affect. Neurosci..

[34] Gliga T, Elsabbagh M, Andravizou A, Johnson M (2009). Faces attract infants’ attention in complex displays. Infancy.

[35] Cousijn J, Hessels RS, der Stigchel SV, Kemner C (2017). Evaluation of the psychometric properties of the gap-overlap task in 10-month-old infants. Infancy.

[36] Leppänen J, Forssman L, Kaatiala J, Yrttiaho S, Wass S (2015). Widely applicable MATLAB routines for automated analysis of saccadic reaction times. Behav. Res. Methods.

[37] Pyykkö Juha, Forssman Linda, Maleta Kenneth, Ashorn Per, Ashorn Ulla, Leppänen Jukka M. (2018). Early development of visual attention in infants in rural Malawi. Developmental Science.

[38] Rose SA, Feldman JF, Jankowski JJ (2012). Implications of infant cognition for executive functions at age 11. Psychol. Sci..

[39] Bedford R, Pickles A, Sharp H, Wright N, Hill J (2015). Reduced face preference in infancy: a developmental precursor to callous-unemotional traits?. Biol. Psychiatry.

[40] Forssman L (2017). Eye-tracking-based assessment of cognitive function in low-resource settings. Arch. Dis. Child..

[41] Atkinson J, Hood B, Wattam-Bell J, Braddick O (1992). Changes in infants’ ability to switch visual attention in the first three months of life. Percept..

[42] Hood BM, Atkinson J (1993). Disengaging visual attention in the infant and adult. Infant Behav. Dev..

[43] Kulke L, Atkinson J, Braddick O (2015). Automatic detection of attention shifts in infancy: eye tracking in the fixation shift paradigm. PLoS One.

[44] The MathWorks Inc., Natick, MA, USA. MATLAB R2012b (2012).

[45] Brainard DH (1997). The psychophysics toolbox. Spatial Vis..

[46] Papageorgiou KA (2014). Individual differences in infant fixation duration relate to attention and behavioral control in childhood. Psychol. Sci..

[47] StataCorp, College Station, TX, USA. Stata Statistical Software: Release 15 (2017).

[48] JASP Team. JASP (Version 0.10.2)[Computer software] (2019).

[49] Jeffreys, H. *Theory of Probability*, 3rd ed., 290 (Clarendon Press, Oxford, 1961).

