# Supplementary material for: Cross-cultural analysis of attention disengagement times supports the dissociation of faces and patterns in the infant brain
Source: Sci Rep. 2019 Oct 8;9:14414. doi: 10.1038/s41598-019-51034-x (PMC6783433; doi:10.1038/s41598-019-51034-x)
Supplement: Supplementary file 1 — Supplementary Information [file 41598_2019_51034_MOESM1_ESM.pdf]

SUPPLEMENTARY INFORMATION: Cross-cultural analysis of attention disengagement times supports the dissociation of faces and patterns in the infant brain

Juha Pyykkö, Per Ashorn, Ulla Ashorn, Dana J. H. Niehaus, & Jukka M. Leppänen

SUPPLEMENTARY METHODS

Analysis of disengagement times

The disengagement time (DT) was defined as the time interval starting at the onset of the lateral stimulus and extending until the point of gaze shifted from the central to the lateral stimulus or a time-out period of 3,500 ms was reached (i.e., censored DTs). In previous studies, DTs have been aggregated by calculating an average of the DTs (e.g.[1–3]), an average of logarithmized DTs as proposed by Csibra *et al.*[4] (e.g.[5–7]), or a median of DTs (e.g.[8]). Sometimes DTs have been aggregated with the inclusion of censored DTs (i.e., trials without gaze shift by the set cut-off value, e.g.[9]), or by calculating the proportion of observed gaze shifts out of the total number of valid trials (e.g.[10,11]). The first approach is problematic given the fact that infants' DTs are characterized by a heterogeneous distribution of quick, delayed and censored values[12,13]. The second approach is a more justified for summarizing heterogeneous distributions, but this approach is complicated by a lack of a clear definition of the distinct population of responses. Consequently, classification of responses into "shift" and "no-shift" trials has been based on arbitrary and variable cut-off value, typically around 1,000 ms. A summary of the aggregating methods used in previous studies is listed in Table S1.

We used a data-driven method to recode the DT data into a binary variable that indicated whether a quick disengagement from the central stimulus occurred or not on a given trial. The distribution of DTs for faces in the current datasets consisted of a primary density of "quick" responses (corresponding to the typical latency of disengagement in infants, approximately 0-1,000 ms), a smaller population of "delayed" responses (approximately 1,000-3,500 ms), and a variable number of censored responses (3,500 ms) (Fig. 2, Table 1). To find the boundaries of these populations for the different datasets, we used unsupervised agglomerative hierarchical clustering to classify the distribution of DTs into distinct groups of observations. The calculation procedure included the following steps: (1) clustering all valid DTs in the face condition that were below 3,500 ms with Ward's method using Euclidean distance, (2) stop clustering when two clusters (groups) were reached, (3) selecting a time point splitting DTs into two separate groups of responses (i.e., a mean of the two values, one from both groups defined in clustering, that were closest to each other), and (4) verifying the clustering outcome by fitting finite mixture models of two distributions to log-transformed DTs.

Hierarchical clustering split non-censored DTs on the face condition into two groups. The cut-off value for quick responses was 1,066 ms for Finland, 833 ms for Malawi 1, 1,110 ms for Malawi 2, and 941 ms for South Africa. In a verification with finite mixture modelling for two distributions, cut-offs were 700 ms for Finland, 883 ms for Malawi 1, 1,067 ms for Malawi 2, and 533 ms for South Africa. Using the cut-off from the primary clustering, we recoded all trials in the data into "quick" and "delayed" disengagements, with the latter category also including censored values.

References

1. Alahyane, N. *et al.* Development and learning of saccadic eye movements in 7- to 42-month-old children. *J. Vis.* **16**, 6, DOI: 10.1167/16.1.6 (2016).

2. Elison, J. T. *et al.* White matter microstructure and atypical visual orienting in 7-month-olds at risk for autism. *Am. J. Psychiatry* **170**, 899–908, DOI: 10.1176/appi.ajp.2012.12091150 (2013).

3. Kulke, L., Atkinson, J. & Braddick, O. Automatic detection of attention shifts in infancy: Eye tracking in the fixation shift paradigm. *PLoS One* **10**, e0142505, DOI: 10.1371/journal.pone.0142505 (2015).

4. Csibra, G., Hernik, M., Mascaro, O., Tatone, D. & Lengyel, M. Statistical treatment of looking-time data. *Dev. Psychol.* **52**, 521–536, DOI: 10.1037/dev0000083 (2016).

5. Elsabbagh, M. *et al.* Visual orienting in the early broader autism phenotype: disengagement and facilitation. *J. Child Psychol. Psychiatry, Allied Discip.* **50**, 637–642, DOI: 10.1111/j.1469-7610.2008.02051.x (2009).

6. Matsuzawa, M. & Shimojo, S. Infants' fast saccades in the gap paradigm and development of visual attention. *Infant Behav. Dev.* **20**, 449–455, DOI: 10.1016/S0163-6383(97)90035-7 (1997).

7. Nakagawa, A. & Sukigara, M. Individual differences in disengagement of fixation and temperament: Longitudinal research on toddlers. *Infant Behav. Dev.* **36**, 728–735, DOI: 10.1016/j.infbeh.2013.08.001 (2013).

8. der Stigchel, S. V., Hessels, R., van Elst, J. & Kemner, C. The disengagement of visual attention in the gap paradigm across adolescence. *Exp. Brain Res.* **235**, 3585–3592, DOI: 10.1007/s00221-017-5085-2 (2017).

9. Leppänen, J., Forssman, L., Kaatiala, J., Yrttiaho, S. & Wass, S. Widely applicable matlab routines for automated analysis of saccadic reaction times. *Behav. Res. Methods* **47**, 538–548, DOI: 10.3758/s13428-014-0473-z (2015).

10. Kataja, E.-L. *et al.* Maternal depressive symptoms during the pre- and postnatal periods and infant attention to emotional faces. *Child Dev.* DOI: 10.1111/cdev.13152 (2018).

11. Yrttiaho, S., Forssman, L., Kaatiala, J. & Leppänen, J. M. Developmental precursors of social brain networks: The emergence of attentional and cortical sensitivity to facial expressions in 5 to 7 months old infants. *PLoS One* **9**, e100811, DOI: 10.1371/journal.pone.0100811 (2014).

12. Papageorgiou, K. A. *et al.* Individual differences in infant fixation duration relate to attention and behavioral control in childhood. *Psychol. Sci.* **25**, 1371–1379, DOI: 10.1177/0956797614531295 (2014).

13. Pyykkö, J. *et al.* Early development of visual attention in infants in rural malawi. *Dev. Sci.* e12761, DOI: 10.1111/desc.12761 (2018).

Study	Age (months)	Lower limit (ms)	Upper limit (ms)	Aggregating method	Includes right-censored values
Alahyane *et al.* (2016)[1]	7-42	50	600	Mean	No
Elison *et al.* (2013)[2]	7	100	1,000	Mean	No
Elsabbagh *et al.* (2009)[5]	9-12	100	1,200	Mean (log)	No
Kataja *et al.* (2018)[10]	8	150	1,000	Binary	Yes
Kulke, Atkinson, & Braddick (2015)[3]	1-9	150	5,000	Mean	No
Leppänen *et al.* (2015)[9]	5-48	150	1,000	Mean	Yes
Matsuzawa & Shimojo (1997)[6]	2.5-12	100	3,000	Mean (log)	No
Nakagawa & Sukigara (2013)[7]	12-36	100	2,600	Mean (log)	No
Van der Stigchel *et al.* (2017)[8]	9-15	0	1,500	Median	No
Yrttiaho, Forssman, Kaatiala, & Leppänen (2014)[11]	5-7	150	1,000	Binary	Yes

Table S1. Lower and upper limits for valid disengagement times in previous studies with infants and young children.

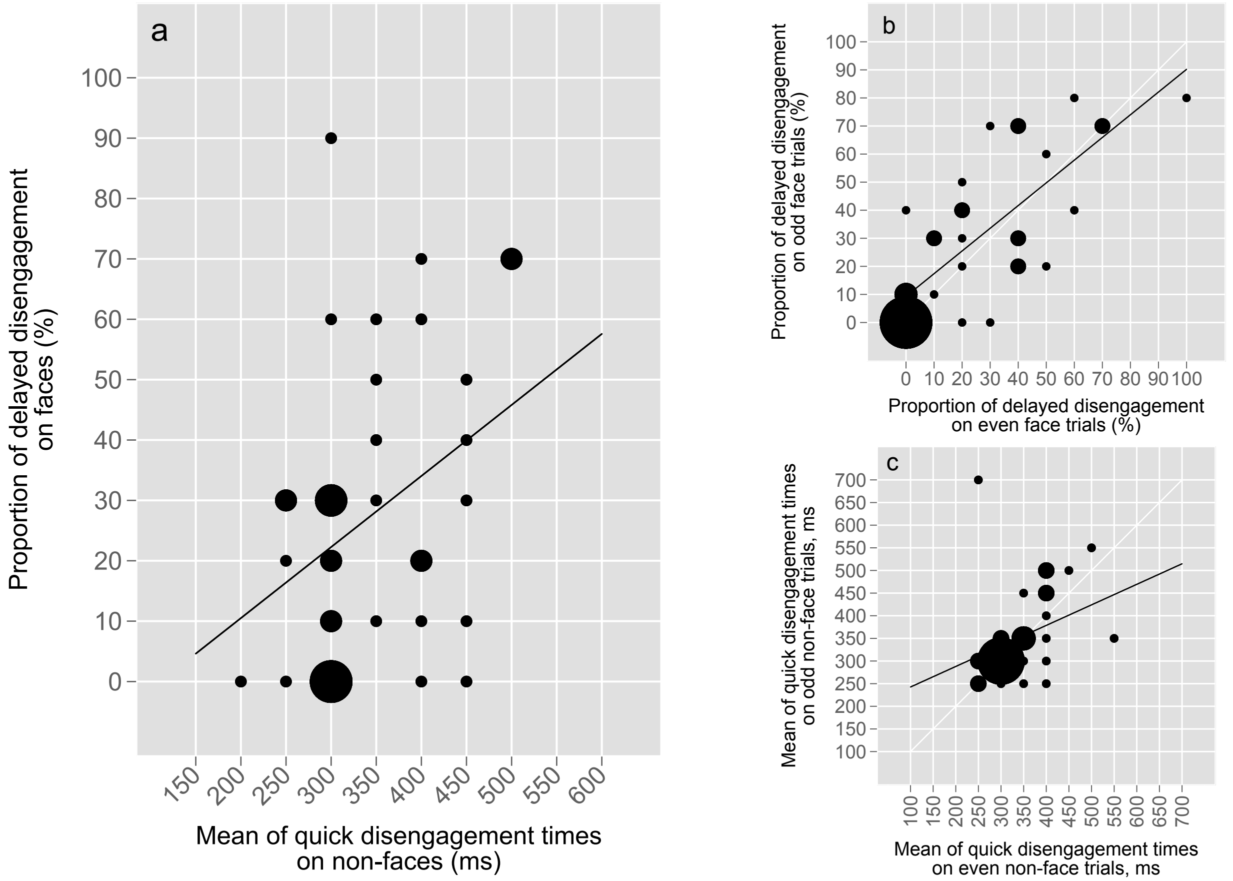

Figure S1. Bubble plots on disengagement data for Finland dataset including participants with ≥ 3 observations for each variable. Circle size is the squared count of participants for each interval. Black line indicates linear fit. (**a**) Covariation of delayed disengagement from faces and oculomotor speed for non-face patterns, $N = 36$, $r_s = 0.37$, $BF_{10} = 2.30$. (**b**) Odd-even split-half reliabilities of delayed disengagement from faces, $N = 35$, $r_s = 0.72$, $BF_{10} > 100$. (**c**) Odd-even split-half reliabilities of oculomotor speed for non-face patterns, $N = 31$, $r_s = 0.45$, $BF_{10} = 4.70$.

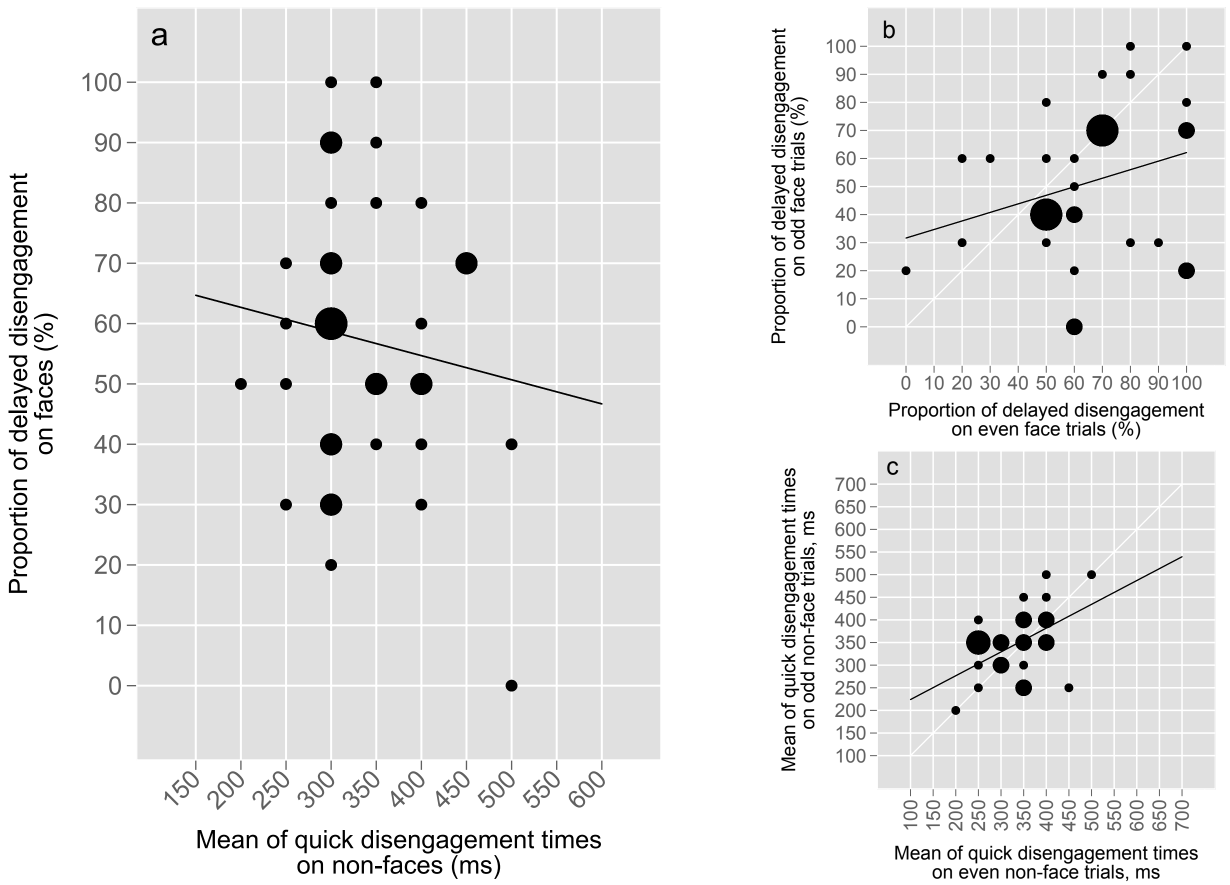

Figure S2. Bubble plots on disengagement data for Malawi 1 dataset including participants with ≥ 3 observations for each variable. Circle size is the squared count of participants for each interval. Black line indicates linear fit. (**a**) Covariation of delayed disengagement from faces and oculomotor speed for non-face patterns, $N = 35$, $r_s = -0.02$, $BF_{10} = 0.21$. (**b**) Odd-even split-half reliabilities of delayed disengagement from faces, $N = 33$, $r_s = 0.28$, $BF_{10} = 0.73$. (**c**) Odd-even split-half reliabilities of oculomotor speed for non-face patterns, $N = 27$, $r_s = 0.53$, $BF_{10} = 10.30$.

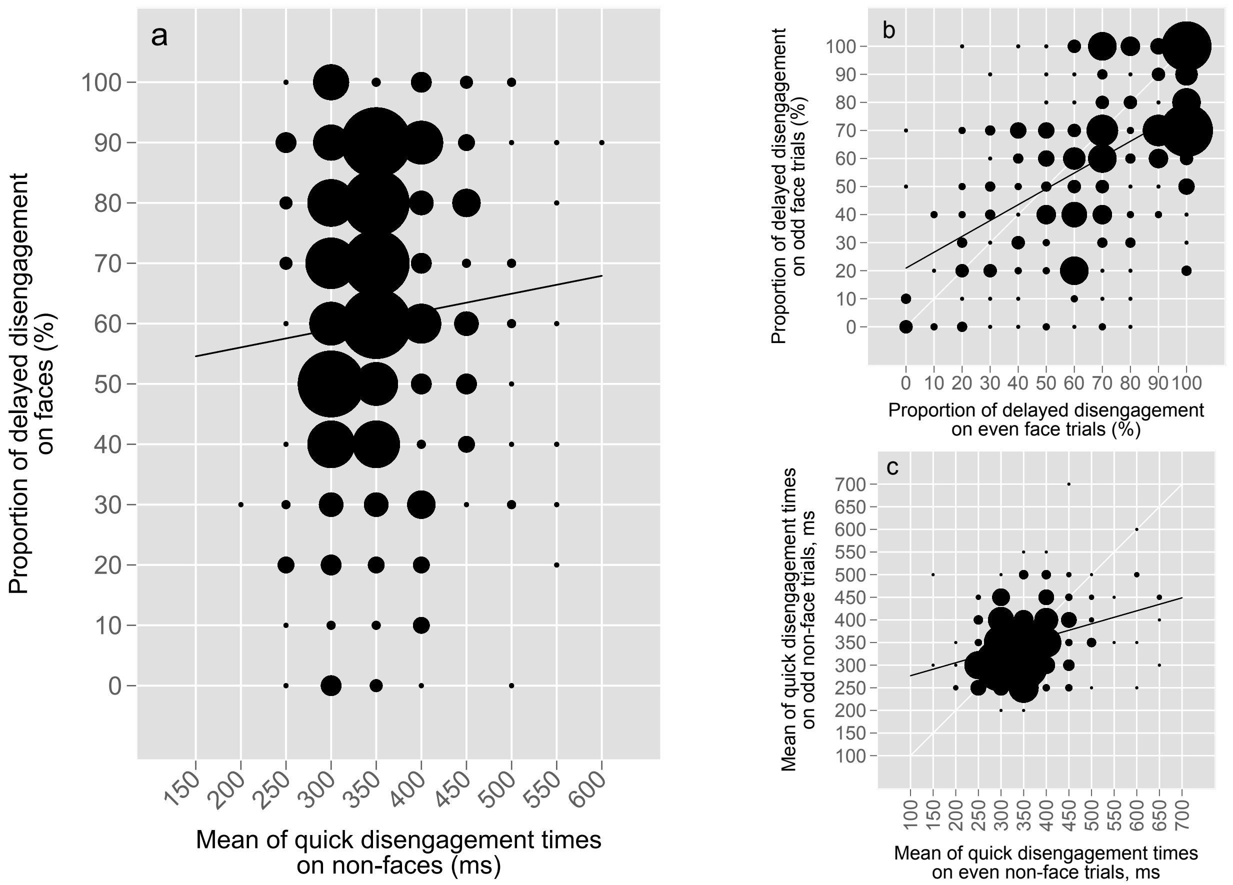

Figure S3. Bubble plots on disengagement data for Malawi 2 dataset including participants with $\geq$ 3 observations for each variable. Circle size is the squared count of participants for each interval. Black line indicates linear fit.(**a**) Covariation of delayed disengagement from faces and oculomotor speed for non-face patterns, N = 345, r_s = 0.08, BF_{10} = 0.18. (**b**) Odd-even split-half reliabilities of delayed disengagement from faces, N = 308, r_s = 0.53, BF_{10} > 100. (**c**) Odd-even split-half reliabilities of oculomotor speed for non-face patterns, N = 273, r_s = 0.29, BF_{10} > 100.

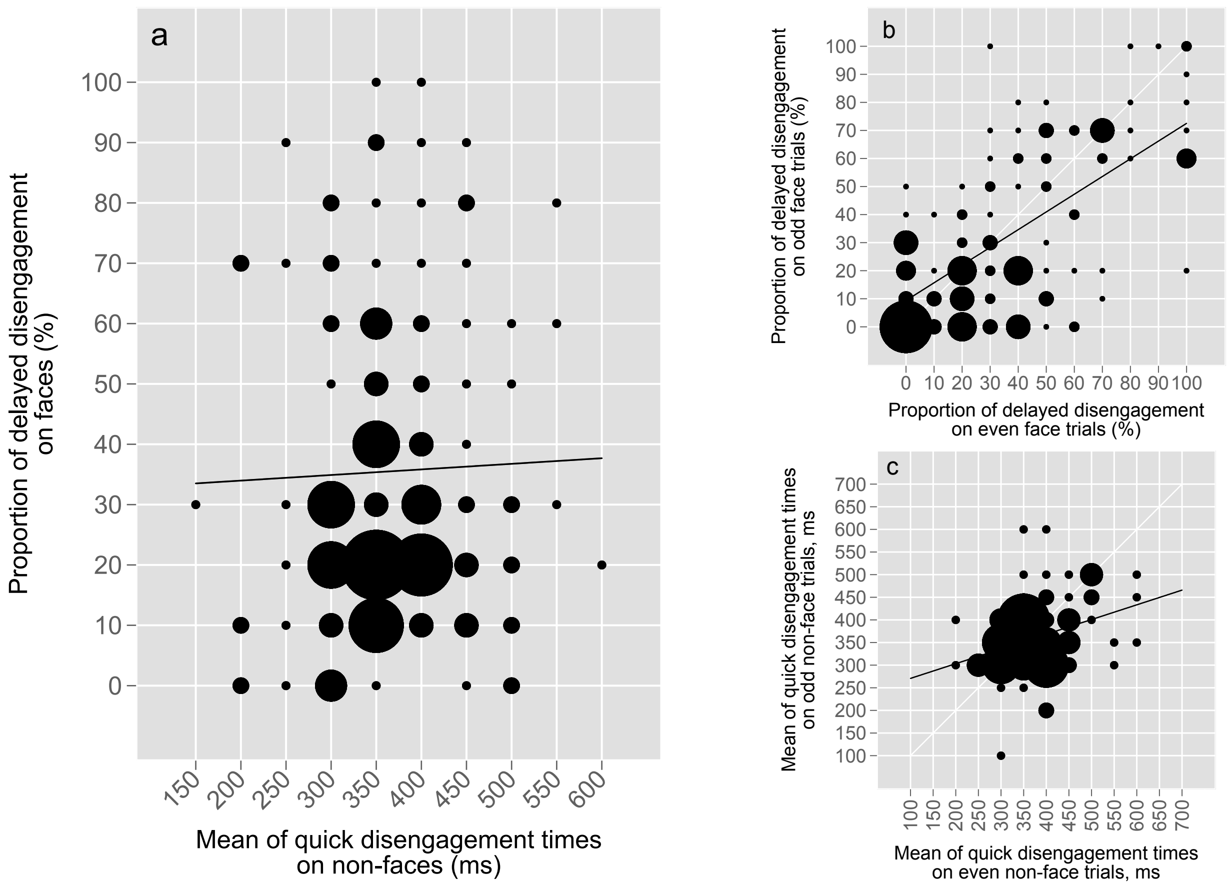

Figure S4. Bubble plots on disengagement data for South Africa dataset including participants with ≥ 3 observations for each variable. Circle size is the squared count of participants for each interval. Black line indicates linear fit. (**a**) Covariation of delayed disengagement from faces and oculomotor speed for non-face patterns, $N = 134$, $r_s = 0.08$, $BF_{10} = 0.16$. (**b**) Odd-even split-half reliabilities of delayed disengagement from faces, $N = 132$, $r_s = 0.57$, $BF_{10} > 100$. (**c**) Odd-even split-half reliabilities of oculomotor speed for non-face patterns, $N = 77$, $r_s = 0.35$, $BF_{10} = 16.43$.
